# Commonly prescribed medicines antagonise anti-MRSA antibiotics and select for resistance

**DOI:** 10.1099/mic.0.001733

**Published:** 2026-07-02

**Authors:** Zoha Sohail, Henry A. Claireaux, Andrew M. Edwards, Edward J.A. Douglas

**Affiliations:** 1 Centre for Bacterial Resistance Biology, Imperial College London, London SW7 2AY, UK; 2 Department of Infectious Disease, Imperial College London, London SW7 2AY, UK; 3 Department of Infectious Disease, King’s College London, London, UK; 4 Academic Department of Military Trauma & Orthopaedics, Research & Clinical Innovation, ICT Centre, Birmingham, UK

**Keywords:** antimicrobial resistance, polypharmacy, prescription medicines, *Staphylococcus aureus*

## Abstract

Many commonly prescribed non-antibiotic medicines have off-target antimicrobial activity, yet their impact on antibiotic efficacy remains poorly understood. In this study, we investigated eight widely used UK prescription medicines and identified simvastatin, amlodipine and fluoxetine as growth inhibitory towards methicillin-resistant *Staphylococcus aureus*. These drugs disrupt bacterial membranes, with amlodipine and fluoxetine also triggering stress responses linked to cell wall and membrane damage. Further mechanistic analysis using transposon mutant screening revealed that simvastatin impairs cell wall synthesis by inhibiting the mevalonate pathway. Notably, checkerboard assays demonstrated antagonistic interactions: simvastatin reduced the efficacy of *β*-lactams and vancomycin, amlodipine with vancomycin and daptomycin and fluoxetine with vancomycin activity. Prolonged exposure to these drugs also accelerated resistance development to vancomycin and daptomycin. Together, these findings underscore the potential for commonly prescribed non-antibiotic medicines to undermine antibiotic therapy, warranting further study given the rising *S. aureus* treatment failures.

## Introduction

*Staphylococcus aureus* is a major human pathogen, responsible for over one million global deaths annually [1]. Although commonly carried as a commensal, this opportunistic pathogen can cause a wide spectrum of infections, ranging from mild skin and soft tissue infections to severe invasive infections, such as bacteraemia [2,3]. Treatment options for these infections largely depend on the antibiotic susceptibility of the causative isolate. For methicillin-susceptible *S. aureus* (MSSA) infections, *β*-lactam antibiotics such as oxacillin are the treatment of choice. In contrast, therapeutic options for methicillin-resistant *S. aureus* (MRSA) are more limited, with recommended agents including vancomycin and daptomycin [4,5].

*S. aureus* infections are more common in elderly populations, with incidence increasing markedly with age and peaking in individuals over 80 years [6,8]. Ageing is accompanied by an increased prevalence of comorbidities [9], which often necessitates the long-term use of multiple medications and contributes to both increased susceptibility to infection and poorer clinical outcomes [10,11]. Specifically, bacteraemia incidence is higher in this patient group and is associated with over twofold increase in mortality and is more likely to progress to complicated and chronic infection types such as osteomyelitis and infective endocarditis, compared to younger adults [12,16]. Reflecting this greater burden of disease, patients with comorbidities receive a disproportionate number of antibiotic prescriptions [17]. In the UK, the presence of comorbidities is associated with a 44% higher rate of antibiotic prescribing in primary care [18]. Conditions most strongly linked to elevated antibiotic use include cardiovascular disease, diabetes, neurological disorders and immunosuppression [19,20]. This increased exposure to antibiotics, combined with frequent healthcare contact, blood tests and invasive interventions, further elevates the risk of infection with drug-resistant pathogens in these populations [21]. Indeed, elderly patients are also more likely to harbour MRSA infections, further complicating treatment and contributing to treatment failure [22].

Non-antibiotic medications prescribed to manage comorbidities have gained interest for potential repurposing, as some demonstrate off-target antimicrobial effects. For example, a large-scale systematic screen of human-targeted drugs against gut bacteria found that ~24% of these non-antibiotic drugs inhibited the growth of at least one bacterial species tested [23]. While many studies have focused on understanding their antimicrobial mechanisms and potential synergy with antibiotics [24,27]. It remains poorly understood whether these medications reduce antibiotic efficacy or select for resistance [21]. Approximately 90% of people >65 take at least one regularly prescribed medicine, with a median of five per patient [20]. Yet, the role of polypharmacy in causally contributing to the high burden of drug-resistant infections and associated morbidity and mortality remains unknown.

Here, we investigate the impact of eight non-antibiotic medicines, commonly prescribed for several indications associated with high antibiotic use [19,28], on the antibiotic susceptibility of MRSA. This addresses a key gap in understanding the impact of commonly used medicines on resistance dynamics.

## Methods

### Bacterial strains and growth conditions

The *S. aureus* strains used in this study are listed in Table 1. *S. aureus* was cultured in 3 ml Mueller–Hinton broth (MHB) and grown overnight at 37 °C with shaking (180 r.p.m.) to reach the stationary phase. When necessary, MHB was supplemented with 10 µg ml^−1^ erythromycin or 90 µg ml^−1^ kanamycin. Since daptomycin requires calcium for activity, laboratory media were supplemented with 1.25 mM CaCl_2_. Prescription non-antibiotics used throughout this study are listed in Table 2. Unless stated otherwise, prescription medications were tested across a twofold dilution series, a standard approach for assessing phenotypes across a broad concentration range. For untreated controls, vehicle controls for the solvent of the relevant prescription medication were included to ensure the solvent did not impact growth.

**Table 1.** Bacterial strains

Strain	Description	Reference
USA300 JE2 (WT)	LAC strain of the USA300 CA-MRSA lineage cured of plasmids	[58]
USA300 JE2 P*vraX-gfp*	USA300 LAC JE2 carrying the P*vraX-gfp* reporter plasmid, Kan^r^	[31]
USA300 JE2 P*dltA-gfp*	USA300 LAC JE2 carrying the P*dltA-gfp* reporter plasmid, Kan^r^	[31]
USA300 JE2 P*recA-gfp*	USA300 LAC JE2 carrying the P*recA-gfp* reporter plasmid, Kan^r^	[32]
USA300 JE2 *crtM*::Tn	USA300 LAC JE2 with a *bursa aurealis* transposon insertion in *crtM*, Ery^r^	[58]
Newman	MSSA, laboratory strain isolated from human infection (CC8), lacks antibiotic-resistant determinants	[89]

### Determination of MICs

The MIC of the prescription non-antibiotics was determined according to the well-established broth microdilution method [29]. In brief, a 96-well microtitre plate was used to prepare a range of drug concentrations in 100 µl of cation-adjusted MHB via a series of twofold serial dilutions. To assess the impact of phospholipids on amlodipine and fluoxetine susceptibility, phosphatidylglycerol (Sigma-Aldrich) was added at a final concentration of 10 µM. For antibiotic synergy determination, checkerboard analyses were performed by preparing twofold serial dilutions of the drugs and antibiotics, with each dilution across a different axis, generating an 8×8 matrix to assess the MICs of each antibiotic when used in combination [30]. Stationary-phase bacteria were diluted to 1×10^6^ c.f.u. ml^−1^ in cation-adjusted MHB, and 100 µl was used to seed each well of the microtitre plate to give a final inoculation density of 5×10^5^ c.f.u. ml^−1^. Plates were then incubated statically at 37 °C for 18 h under ambient air conditions, at which point the MIC was defined as the lowest antibiotic concentration at which no visible bacterial growth was observed. Where checkerboard analysis was performed, OD was measured at _600nm_ using a Bio-Rad iMark microplate absorbance reader (Bio-Rad Laboratories). Where growth curves are presented, plates were incubated at 37 °C with continuous shaking in a microplate reader (Tecan Spark, MIC9412), and OD_600nm_ was recorded every 10 min for 16 h.

### Fluorescent reporter assays

Promoter-*gfp* constructs were used to measure gene expression in response to amlodipine, simvastatin and fluoxetine, according to a pre-established protocol [31,32]. In brief, amlodipine, simvastatin and fluoxetine were serially diluted 1:2 in 100 µl of cation-adjusted MHB (P*vraX-gfp,* P*recA-gfp*) or Rosewell Park Memorial Institute 1640 (RPMI) (Gibco, USA) (P*dltA-gfp*) in a black, clear-bottom 96-well plate (Greiner, Germany). Additionally, DMSO (Sigma-Aldrich) was added to all wells in the simvastatin dilution series at a final concentration of 2% to maintain drug solubility. Positive controls were run in parallel using vancomycin for *PvraX-gfp*, ciprofloxacin for *PrecA-gfp* and polymyxin B for P*dltA-gfp*. Overnight reporter cultures were diluted to a final concentration of 10^8^ c.f.u. ml^−1^ in MHB or RPMI 1640, and 100 µl of the diluted cultures was added to each well. The plates were placed in a microplate reader (Tecan Spark, MIC9412) at 37 °C with shaking, and GFP fluorescence (excitation 475 nm, emission 525 nm), and OD_600nm_ was measured every 15 min for 16 h. Raw fluorescence readings were normalized according to the OD_600nm_ readings.

### Determination of membrane permeability

To measure membrane permeabilization by amlodipine, simvastatin and fluoxetine, the well-established SYTOX green assay was used [33,34]. Stationary-phase *S. aureus,* at an inoculum density of 1×10^8^ c.f.u. ml^−1^ was added to 3 ml of MHB containing the relevant prescription antibiotics across a 1:2 dilution series. The fluorescent probe SYTOX green (Invitrogen) was added to these cultures at a final concentration of 1 µM. Aliquots (200 µl) were transferred to a black microtitre plate with clear-bottomed wells (Greiner Bio-One). Fluorescence and OD_600nm_ were measured in a Tecan Infinite 200 Pro plate reader (excitation at 535 nm, emission at 617 nm) every 15 min for 2 h at 37 °C with shaking. Raw fluorescence readings were normalized according to OD_600nm_. OD_600nm_ measurements were also used to assess the extent of cell lysis following exposure to these drugs using the following equation, where OD_600nm_ t*n* indicated the OD_600nm_ at a given time point and OD_600nm_ t*0* indicated the OD_600nm_ at time 0 min:


$$Percentage\;lysis=100-\left(100\;\times \left(\frac{OD600nm\;tn}{OD600nm\;t0}\right)\right)$$

### Screening of the NTML for simvastatin resistance determinants

The Nebraska Transposon Mutant Library (NTML) comprises 1,920 individual mutants arrayed across five 384-well plates, which were used to screen for mutants conferring simvastatin resistance. Mutants from each of the five plates were used to inoculate twenty 96-well microtiter plates, with each well containing MHB supplemented with 10 µg ml^−1^ of erythromycin (Sigma-Aldrich) to maintain selection for the transposon insertion. These 96-well plates were incubated statically at 37 °C overnight. For storage, the NTML library was stored in 25% glycerol at −80 °C. For the screening assay, square agar plates containing MHB were prepared with simvastatin at 1× simvastatin MIC (25 µg ml^−1^). After overnight growth, cultures from each well of the 96-well plates were spotted (3.5 µl) on the simvastatin agar plates. Plates were again statically incubated overnight at 37 °C. Mutant identities were determined by cross-referencing the NTML plate map.

### Staphyloxanthin pigment analysis

A 3 ml overnight culture of *S. aureus* JE2 and the control strain JE2 *crtM*::Tn was prepared in MHB. The *S. aureus* JE2 MHB culture was supplemented with varying concentrations of simvastatin (0 µg ml^−1^, 3.125 µg ml^−1^, 6.25 µg ml^−1^ and 12.5 µg ml^−1^), with and without the addition of mevalonate (90469, Sigma-Aldrich) at a concentration of 100 µM. The following day, 1 ml of the overnight cultures was centrifuged at 10,000 ***g*** for 1 min. The pellets were then resuspended in 200 µl of methanol and incubated at 55 °C in a water bath for 30 min. Following incubation, the pellets were centrifuged at 10,000 ***g*** for 1 min. The supernatants were recovered, and the absorbance was read at 453 nm (Tecan Spark, MIC9412). A methanol-only control was included as a blank.

### Serial passaging with antibiotics

A single colony of *S. aureus* JE2 was grown overnight in 3 ml MHB. Two 96-well plates were prepared, one for vancomycin and one for daptomycin passaging. In brief, a 96-well microtitre plate was used to prepare a range of daptomycin and vancomycin concentrations in 100 µl of cation-adjusted MHB via a series of twofold serial dilutions. Following this, stationary-phase bacteria were diluted to 1×10^6^ c.f.u. ml^−1^ in cation-adjusted MHB, with and without simvastatin, amlodipine or fluoxetine at a final concentration of 12.5 µg ml^−1^ and 100 µl used to seed each well of the microtitre plate to give a final inoculation density of 5×10^5^ c.f.u. ml^−1^. Plates were then incubated statically at 37 °C for 18 h under ambient air conditions. Each day, bacteria from the well immediately adjacent to the MIC (the highest concentration in which there was observable growth) were diluted 1:5,000 in fresh MHB and plated on a new microtitre prepared as above. Serial passaging was performed for 20 days, with daily OD_600nm_ measurements (Tecan Spark, MIC9412) used to monitor changes in MIC in the presence of each drug. To confirm that changes in OD_600nm_ were not a result of contamination, aliquots from each well were diluted tenfold in sterile PBS and spotted on MHBA. This was followed by an additional 5-day passage in the absence of simvastatin, amlodipine and fluoxetine to assess the stability of the resistance phenotype.

### Statistical analysis

The data are derived from three biologically independent experiments unless stated otherwise. The data are presented as the mean of the biological repeats with error bars representing the standard deviation from the mean. Where appropriate, statistical analysis is performed using an ordinary one-way or two-way ANOVA as stated. Details of the post hoc comparison and the asterisks representing significance levels are stated in the figure legends. All statistical analyses were performed using GraphPad Prism software Version 10.5.0.

## Results

### Simvastatin, fluoxetine and amlodipine exhibit anti-MRSA antimicrobial activity

**Fig. 1.**
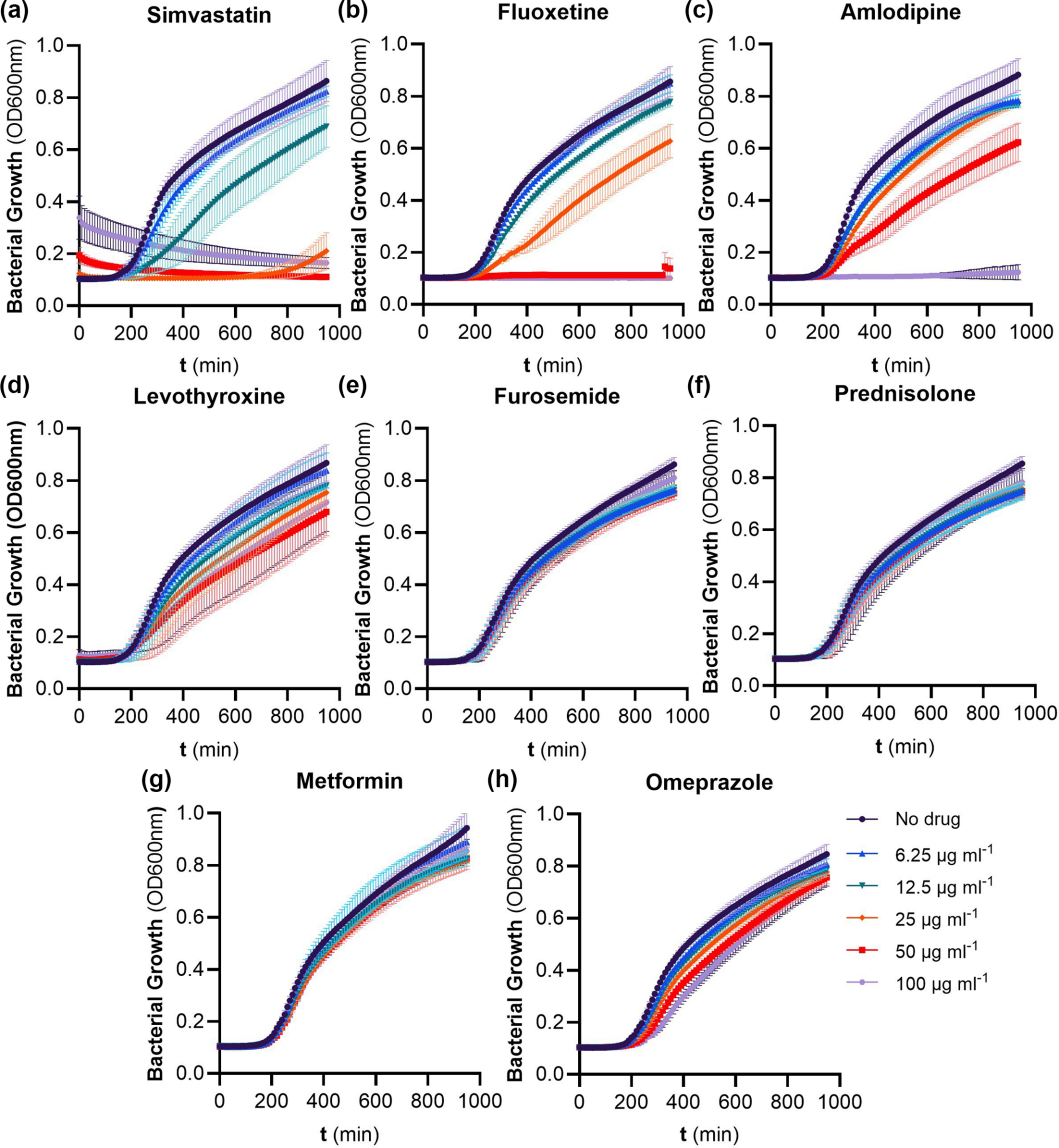
Simvastatin, amlodipine and fluoxetine have antimicrobial activity. (a–h) 16-h growth curves of *S. aureus* JE2 in the presence of a 1:2 dilution series of different prescription non-antibiotics. All experiments were replicated in *n*=3 independent assays. Error bars show the standard deviation of the mean. Note that the high initial starting optical density of simvastatin at 100 µg ml^−1^ is due to this concentration being close to the solubility threshold of this compound. Significant differences were determined between the no-drug condition and treated conditions via two-way ANOVA (Table S1).

Eight commonly prescribed non-antibiotic medicines commonly prescribed for several indications, covering diverse drug classes, were chosen for their high incidence of use [28]. For example, it is estimated that >7 million people in the UK take statins [35]. A summary of these medicines, their indications and their incidence of use is provided in Table S1 (available in the online Supplementary Material). To determine whether these prescription non-antibiotic medicines exhibited antimicrobial activity, we monitored bacterial growth over 16 h in the presence of a twofold dilution series of each drug (Fig. 1a-h). Among the compounds tested, simvastatin, fluoxetine and amlodipine showed notable activity against *S. aureus*, with MICs of 25, 50 and 100 µg ml^−1^, respectively, which is in keeping with previous findings (Fig. 1a, b and c) [36,38]. Although we were unable to determine an MIC for levothyroxine and omeprazole, we noted a dose-dependent delay in growth rate in the presence of these drugs (Fig. 1d and h). Together, this confirms that a subset of prescription non-antibiotic medicines possesses antimicrobial activity against *S. aureus*.

### Prescription non-antibiotics activate *S. aureus* stress responses

**Fig. 2.**
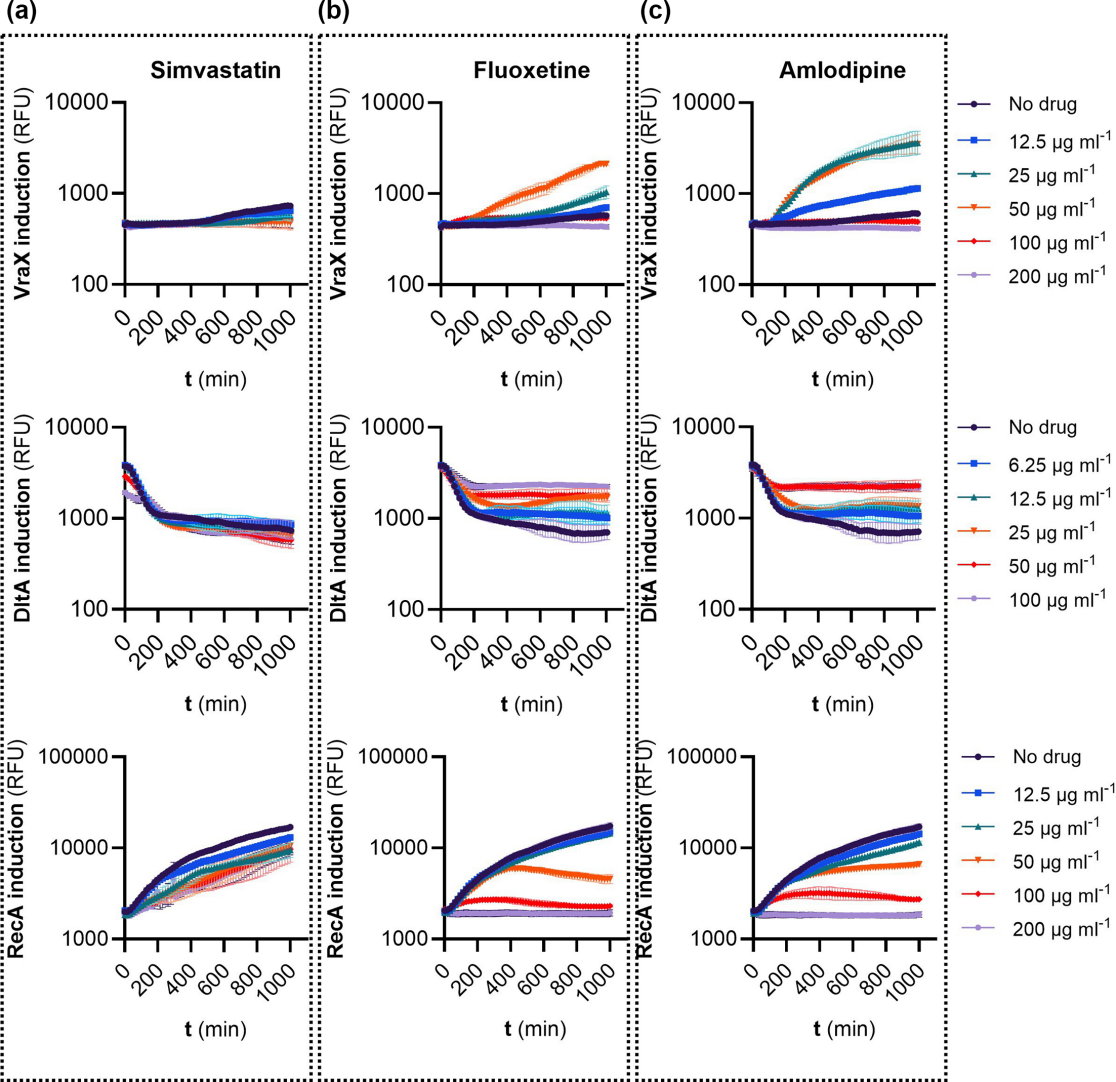
Fluoxetine and amlodipine activate the cell wall and membrane stress responses. (**a**) Induction assays of p*vraX-*GFP, p*dltA-*GFP and p*recA-*GFP in the presence of a 1:2 dilution series of simvastatin as determined by GFP accumulation over time. (**b**) Induction assays of p*vraX-*GFP, p*dltA-*GFP and p*recA-*GFP in the presence of a 1:2 dilution series of fluoxetine, as determined by GFP accumulation over time. (**c**) Induction assays of p*vraX-*GFP, p*dltA-*GFP and p*recA-*GFP in the presence of a 1:2 dilution series of amlodipine as determined by GFP accumulation over time. Relative fluorescence units (RFU) were normalized according to OD_600nm_ values to account for changes in bacterial viability. All experiments were replicated in *n*=3 independent assays in *S. aureus* JE2. Error bars show the standard deviation of the mean. Significant differences were determined between the no drug condition and treated conditions via two-way ANOVA (Table S2).

*S. aureus* has several dedicated signalling pathways, which are used to sense and respond to environmental stimuli such as the presence of antibiotics [39], particularly VraSR (cell wall damage) [40,41], GraSR (membrane damage) [42] and SOS (DNA damage) [43,44]. Therefore, to better understand the antimicrobial activity of simvastatin, fluoxetine and amlodipine, we tested whether they activated these key stress pathways. To achieve this, we used fluorescent reporter systems in which *gfp* expression was driven by the promoter of a gene activated by the relevant stress pathway. For the VraSR, GraSR and SOS pathways, the promoters *vraX*, *dltA* and *recA* were used, respectively, as they are well-established targets of activation by each pathway [31,32]. A dose-dependent increase in *vraX* expression was observed in response to fluoxetine, amlodipine and the positive control antibiotic vancomycin, confirming that these compounds induce cell wall stress (Figs 2b, c and S1a). However, activation of *vraX* expression was not detected in response to simvastatin (Fig. 2a). Similarly, *dltA* expression increased in a dose-dependent manner in response to fluoxetine, amlodipine and the control antibiotic polymyxin B, but not simvastatin, indicating that the former compounds also induce membrane damage (Figs 2a, b, c and S1b). In contrast, neither of the three non-antibiotics activated *recA* expression, unlike the positive control antibiotic ciprofloxacin (Figs 2a, b, c and S1c).

**Table 2.** Prescription non-antibiotic drugs

Non-antibiotic drug	CAT no.	Solvent
Simvastatin	PHR1438	DMSO
Prednisolone	PHR1043	Ethanol
Furosemide	PHR1057	Methanol
Fluoxetine hydrochloride	PHR1394	Methanol
Amlodipine besylate	PHR1185	Ethanol
Levothyroxine	PHR1613	DMSO
Omeprazole	PHR1059	Ethanol
Metformin	PHR1084	Water

### Prescription non-antibiotic medicines damage the *S. aureus* membrane

**Fig. 3.**
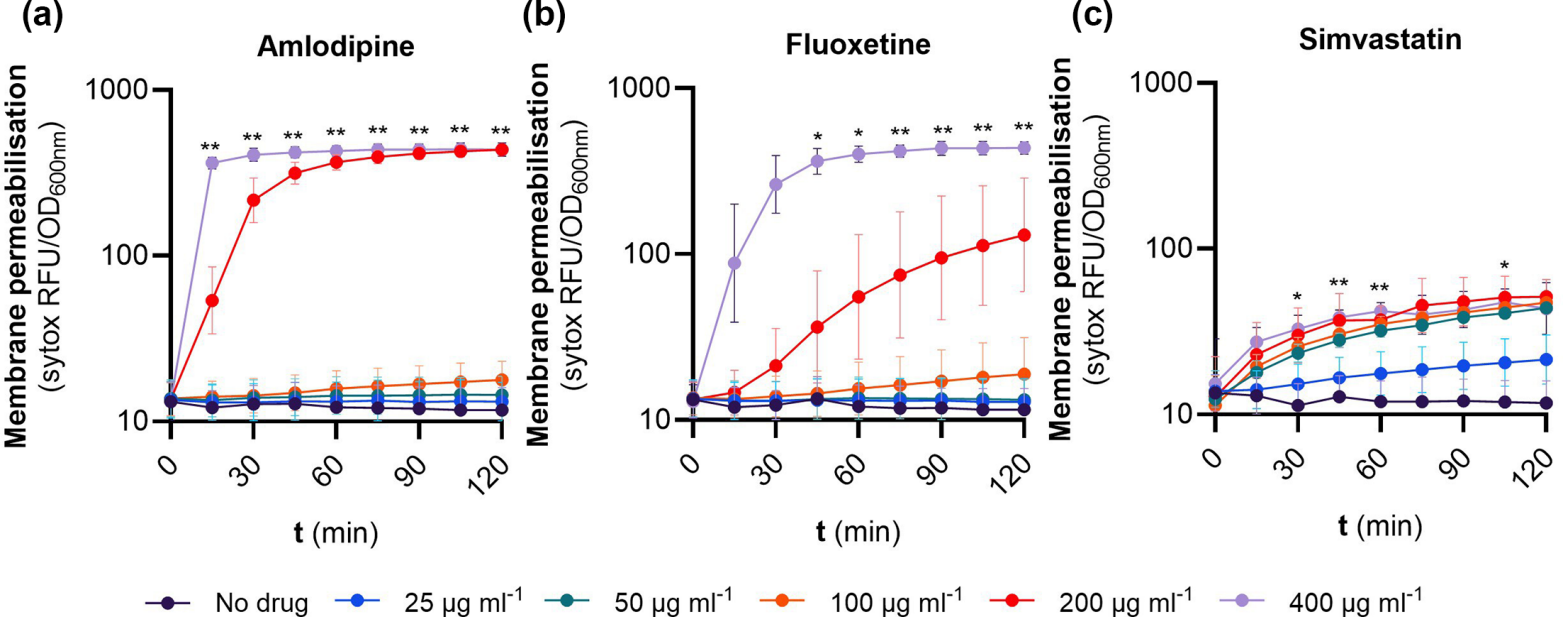
Amlodipine, fluoxetine and simvastatin permeabilize the membrane. (**a, b, c**) Membrane disruption of *S. aureus* JE2 exposed to a 1:2 dilution series of (**a**) amlodipine, (**b**) fluoxetine and (**c**) simvastatin, for 2 h, as determined by uptake of the fluorescent nucleic acid dye Sytox green. Relative fluorescence units (RFU) were normalized according to OD_600nm_ values to account for changes in bacterial viability. Significant differences were determined between the no drug condition and the 400 µg ml^−1^ treated condition of each drug by two-way repeated measures ANOVA with post hoc Dunnett’s test to correct for multiple comparisons (Table 3). *P<0.05; **P<0.01; the absence of an asterisk indicates a non-significant difference.

Antimicrobials that simultaneously activate both cell wall and membrane stress responses are typically associated with a direct membrane-damaging mechanism of action, such as daptomycin [45,47]. Therefore, to determine whether these prescription non-antibiotics also damage the bacterial membrane, we measured the ingress of the cell-impermeant nucleic acid stain Sytox Green in JE2 cells exposed to a 1:2 dilution series of amlodipine, fluoxetine or simvastatin. Consistent with a direct membrane-damaging mechanism of action, both amlodipine and fluoxetine rapidly permeabilized the membrane, as evidenced by saturation of the Sytox fluorescence by 45 min (Fig. 3a and b). This rapid Sytox accumulation was associated with mild, dose-dependent cell lysis, as calculated via changes in OD_600nm_ over time, further supporting the membrane-damaging activity of these drugs against *S. aureus* (Fig. S2a and b). Both amlodipine and fluoxetine are cationic, with amlodipine bearing a primary amine and fluoxetine a secondary amine. Therefore, we hypothesized that the membrane damage caused by these drugs was mediated through electrostatic interactions with negatively charged phospholipids in the *S. aureus* membrane. In support of this hypothesis, supplementation of the MHB with 10 µM of the negatively charged phosphatidylglycerol lipid increased the MICs of amlodipine and fluoxetine fourfold, to 400 and 200 µg ml^−1^, respectively (Table S2).

Simvastatin exposure resulted in a slower and less pronounced accumulation of Sytox (Fig. 3b). This delayed kinetic profile is typical of antimicrobials that perturb phospholipid biosynthesis or other upstream biosynthetic processes, which indirectly compromise the membrane or cell envelope and weaken barrier function [48,50]. Supporting this, proteomic and macromolecular-synthesis studies have shown that simvastatin inhibits multiple biosynthetic processes, including DNA, RNA, protein, cell wall and lipid synthesis [51].

### Transposon mutant screening reveals pleiotropic determinants of simvastatin resistance

**Fig. 4.**
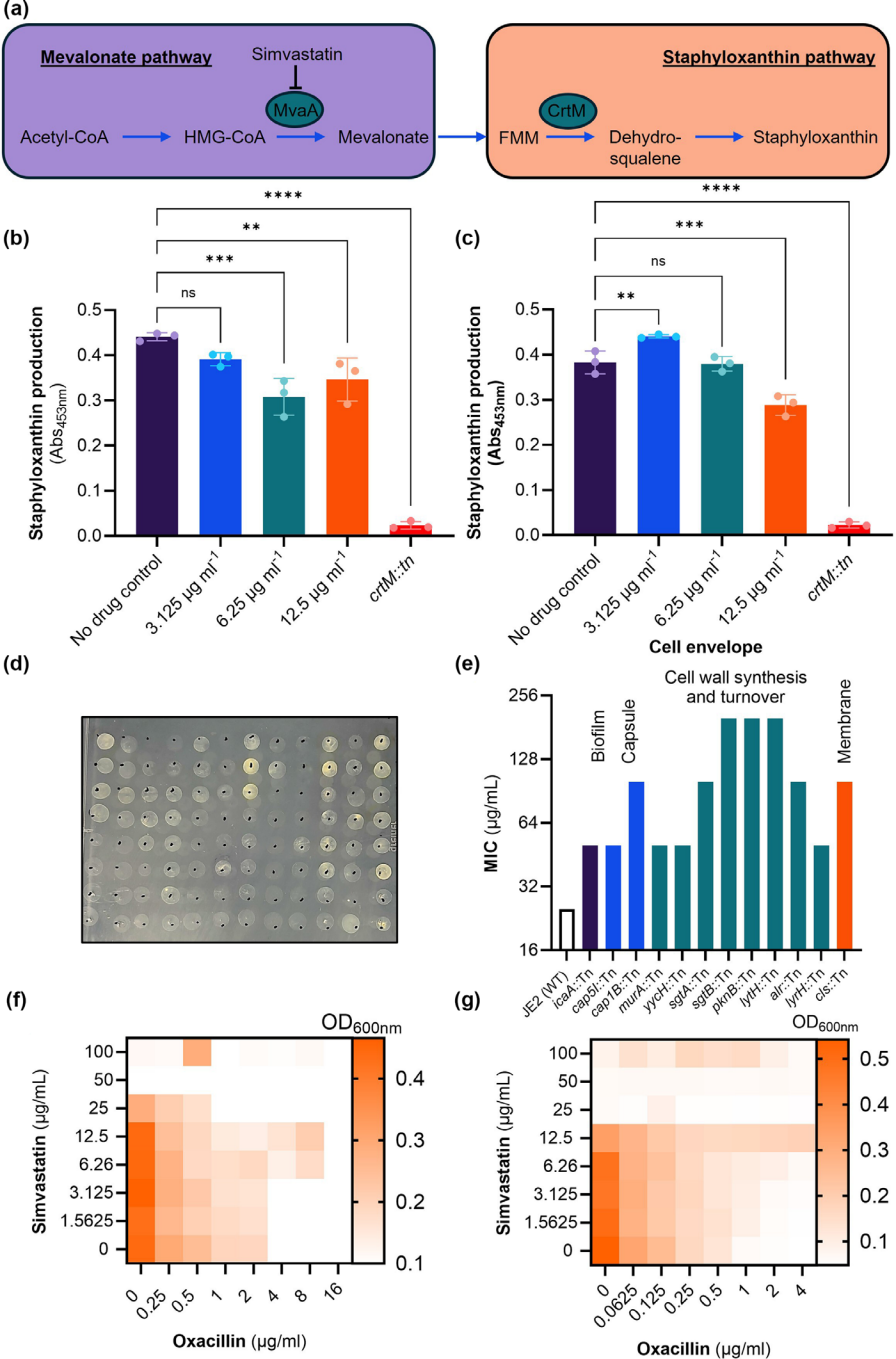
Screening of the NTML reveals pleiotropic routes for simvastatin resistance. (**a**) Summary diagram of how the mevalonate pathway feeds into staphyloxanthin production. Acetyl-CoA is converted to 3-hydroxy-3-methylglutaryl-CoA (HMG-CoA). This is subsequently converted to mevalonate by MvaA, which is inhibited by simvastatin. A multi-step reaction converts mevalonate into farnesyl pyrophosphate (FPP). FPP feeds into peptidoglycan, lipid and staphyloxanthin synthesis. CrtM catalyses the production of dehydrosqualene from FPP, ultimately resulting in the synthesis of staphyloxanthin. (**b**) Staphyloxanthin abundance in *S. aureus* JE2 exposed, or not, to 3.125, 6.25 or 12.5 µg ml^−1^ simvastatin overnight. (**c**) Staphyloxanthin abundance in *S. aureus* JE2 exposed, or not, to 3.125, 6.25 or 12.5 µg ml^−1^ simvastatin overnight, in the presence of exogenous mevalonate 10 µg ml^−1^. JE2 *crtM::*Tn is used as a negative control. (**d**) A representative plate from the NTML transposon screen. (**e**) MIC of confirmed transposon mutant hits defective in cell envelope synthesis. (**f**) Checkerboard broth microdilution assay showing the antagonistic interaction between simvastatin and oxacillin against *S. aureus* JE2. (g) Checkerboard broth microdilution assay showing the antagonistic interaction between simvastatin and oxacillin against *S. aureus* Newman. Significant differences were determined by one-way repeated measures ANOVA between the no-drug control and simvastatin-treated conditions. *P<0.05; **P<0.01; ***P<0.001; ****P<0.0001; ns, not significant.

Since simvastatin did not activate either of the reporter assays used, we decided to characterize simvastatin in more detail. Simvastatin inhibits HMG-CoA reductase in humans, blocking the rate-limiting step of cholesterol biosynthesis in the mevalonate pathway [52]. In *S. aureus*, it concomitantly inhibits the bacterial HMG-CoA reductase homologue MvaA, disrupting isoprenoid, lipid and peptidoglycan synthesis pathways essential for cell viability (Fig. 4a) [51,53,55]. Further demonstrating simvastatin’s effect on isoprenoid synthesis, we and others have shown that it inhibits the production of the isoprenoid-dependent carotenoid pigment staphyloxanthin (Fig. 4b) [56,57]. Supplementation of exogenous mevalonate reversed the inhibitory activity of simvastatin on staphyloxanthin production, confirming its inhibitory activity on the isoprenoid synthesis pathway (Fig. 4). To further elucidate simvastatin’s effects on *S. aureus* and identify genes and pathways that confer resistance, we screened the NTML for mutants exhibiting reduced susceptibility at 1× MIC of simvastatin in Mueller–Hinton broth agar (Fig. 4d) [58]. The 101 mutants that grew at this concentration were tested for reduced simvastatin susceptibility by broth MIC testing, yielding 77 validated hits (Supplementary Table 2). Of these hits, 12 were associated with cell envelope synthesis (Fig. 4e), 16 with DNA and protein synthesis (Table S3) and 15 with metabolism and redox (Table S3), highlighting the pleiotropic effect of inhibiting isoprenoid synthesis in *S. aureus*. The remaining hits (Table S3) were associated with virulence, transport, phage-associated genes or had unknown function. Notably, several identified genes have previously been linked to altered susceptibility to cell wall antibiotics, including *β*-lactams (*murA*, *sgtA*, *sgtB*, *yycH*, *pknB*, *lytH*, *alr*, *clpC*) [59,65], vancomycin (*alr*, *yycH*, *sgtA*, *sgtB*, *clpC*) [60,63, 66] and daptomycin (*cls*, *clpC*, *yycH*) [63,66, 67]. This overlap suggests that simvastatin’s inhibition of isoprenoid biosynthesis induces cell envelope damage analogous to these agents, potentially selecting for cross-resistance mutations while also creating opportunities for synergy.

Simvastatin has previously been shown to synergize with *β*-lactams against MRSA via time kill analysis [68]. Statins’ synergy arises from their disruption of fluid membrane microdomains enriched in isoprenoid lipids, thereby impairing PBP2a oligomerization, which is essential for methicillin resistance [68]. To further test the potential for simvastatin to synergize with *β*-lactams, we performed a checkerboard broth microdilution assay (Fig. 4e). Unlike previously [68], we observed high levels of antagonism between oxacillin and simvastatin against JE2, with a fractional inhibitory concentration index (FICI) of >4 (Fig. 4f). In the presence of sub-inhibitory concentrations of simvastatin, the oxacillin MIC shifted fourfold to 16 µg ml^−1^ (Fig. 4f). To test whether this phenotype extended to additional strains, we repeated the checkerboard analysis for the MSSA strain Newman (Fig. 4g). Similarly, simvastatin antagonized oxacillin susceptibility with a FICI >4. Furthermore, in the presence of 12.5 µg ml^−1^, the oxacillin MIC shifted from 1 µg ml^−1^ (susceptible) to 4 µg ml^−1^ (resistant), according to CLSI breakpoints. Subsequent MIC testing of bacteria grown from these wells in the absence of simvastatin reverted the MIC to 1 µg ml^−1^, potentially indicating that simvastatin may confer transient *β*-lactam resistance independent of PBP2A, more specifically, borderline oxacillin-resistant *S. aureus* (BORSA).

### Prescription non-antibiotic medicines accelerate the emergence of resistance to anti-MRSA antibiotics

**Fig. 5.**
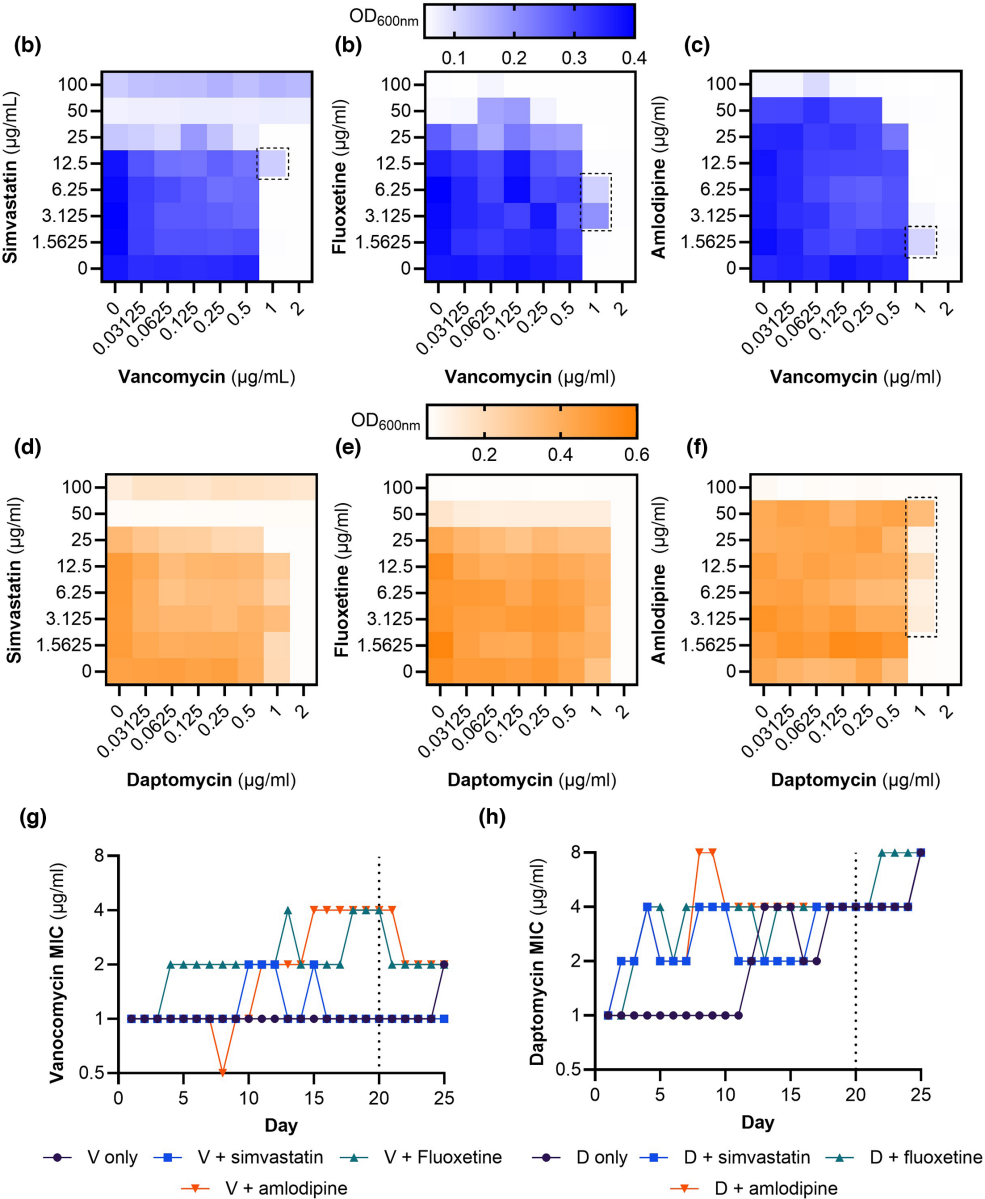
Simvastatin, fluoxetine and amlodipine promote resistance to vancomycin and daptomycin. (**a, b, c**) Checkerboard broth microdilution assay showing the antagonistic interaction between vancomycin and (a) simvastatin, (**b**) fluoxetine and (c) amlodipine against *S. aureus* JE2. (**d, e, f**) Checkerboard broth microdilution assay between daptomycin and (a) simvastatin, (**b**) fluoxetine and (**c**) amlodipine against *S. aureus* JE2. Cells displaying antagonism are highlighted by a dashed square. (**g**) Increase of vancomycin MIC, following daily passage in either vancomycin (**v**) alone or vancomycin in combination with sub-inhibitory simvastatin, fluoxetine or amlodipine. (**h**) Increase of vancomycin MIC, following daily passage in either daptomycin (**d**) alone, or daptomycin in combination with sub-inhibitory simvastatin, fluoxetine or amlodipine. The dashed line at day 20 indicates where co-selection with simvastatin, fluoxetine or amlodipine was removed.

First-line anti-MRSA antibiotics vancomycin and daptomycin activate the VraSR and GraSR stress responses, respectively. Having demonstrated that fluoxetine and amlodipine also activate the VraSR and GraSR stress pathways and that simvastatin disrupts cell wall and membrane synthesis, we assessed whether these drugs could synergize with these antibiotics. Checkerboard broth microdilution assays were conducted to evaluate interactions between vancomycin and each of the following non-antibiotic agents: simvastatin, fluoxetine and amlodipine (Fig. 5a, b c and S3). At sub-inhibitory concentrations, all three compounds reduced bacterial susceptibility to vancomycin, evidenced by an increase in the vancomycin MIC (Fig. 5a, b and c). Checkerboard broth microdilution assays were also conducted between daptomycin and these non-antibiotic agents (Fig. 5d, e, f and S3). No evidence of synergy or antagonism was observed between daptomycin and simvastatin or daptomycin and fluoxetine. However, sub-inhibitory concentrations of amlodipine antagonized daptomycin susceptibility, causing an increase in MIC from 1 µg ml^−1^ to 2 µg ml^−1^ (Fig. 5f). Collectively, these findings suggest that certain non-antibiotic drugs can impair antibiotic efficacy, potentially through shared mechanisms of action or the induction of cellular stress responses.

Sustained activation of the VraSR and GraSR stress response systems has been linked to the development of resistance to vancomycin and daptomycin [69,73]. Given the observed antagonism between the non-antibiotic agents and these antibiotics, together with evidence that the same compounds activate VraSR and GraSR, we hypothesized that these non-antibiotics may impose a selective pressure that promotes the emergence of vancomycin and daptomycin resistance. To test this, JE2 was passaged daily in vancomycin or daptomycin alone or in combination with the non-antibiotic drugs (Fig. 5g and h). When passaged in vancomycin alone, the MIC shifted twofold on the 25th passage (Fig. 5g). However, when vancomycin was combined with either simvastatin, fluoxetine or amlodipine, MIC shifts were observed at earlier passage days (Fig. 5g). A twofold shift in vancomycin MIC was observed at day 4 for the vancomycin–fluoxetine combination, which rose to fourfold (4 µg ml^−1^) at day 18 (Fig. 5g). Similarly, the vancomycin MIC shifted twofold at day 11 for the vancomycin–amlodipine combination, further rising fourfold at day 15 (Fig. 5g). Simvastatin also caused a twofold increase in vancomycin MIC; however, this was unstable and reverted to starting MIC (Fig. 5g). When non-antibiotic co-selection was removed at day 20, the vancomycin MIC decreased twofold, highlighting the selective pressure of these drugs.

Daptomycin serial passage resulted in a twofold increase in MIC at day 12, a fourfold increase at day 18 and an eightfold increase at day 25. In keeping with the antagonistic checkerboard analysis, co-selecting with simvastatin caused a twofold shift in MIC at day 2. The following passage days alternated between two- and fourfold increases in MIC, with a stable fourfold increase in MIC achieved at day 21, and an eightfold increase at day 25. A stable fourfold increase in daptomycin MIC was also achieved at day 14, co-selecting with fluoxetine. An eightfold increase in daptomycin MIC was also achieved earlier at day 22 for this combination. When passaged with amlodipine, the daptomycin MIC shifted sharply by eightfold on day 8, followed by a twofold drop, which was maintained until day 25, where it again rose twofold to 8 µg ml^−1^. Unlike the vancomycin passage, the removal of the co-selecting non-antibiotic drugs did not influence the daptomycin MIC.

## Discussion

Older adults experience disproportionately high rates of drug-resistant infections, which are associated with poor clinical outcomes, including increased morbidity and mortality. Antibiotic use is also markedly elevated in this population, particularly among individuals with multiple comorbidities. Management of these chronic conditions often necessitates long-term pharmacotherapy, with the number of prescribed medications increasing substantially with age. Therefore, in this study, we asked whether this pattern of extensive medication use may contribute to antimicrobial resistance through complex drug interactions or poorly characterized additional selection pressures. We screened eight widely prescribed non-antibiotic medications, spanning therapeutic classes commonly used to manage comorbidities associated with high antibiotic use, for antimicrobial activity against MRSA. Interestingly, three were found to have robust antimicrobial activity: simvastatin, amlodipine and fluoxetine. These three medications were selected for further characterization of the antimicrobial mechanism of action and impact on antibiotic susceptibility, as we hypothesized that medications exhibiting off-target antimicrobial effects were more likely to modulate antibiotic efficacy.

We found that both amlodipine and fluoxetine activated cell wall and membrane stress responses, consistent with mechanisms involving disruption of membrane integrity and interference with cell wall synthesis. Membrane perturbation was further supported and demonstrated by increased accumulation of the fluorescent DNA stain Sytox Green. Beyond *S. aureus*, both agents have demonstrated antimicrobial activity against a range of bacterial species. However, the reduced potency observed in Gram-negative organisms suggests that these compounds likely act at the inner membrane, with activity constrained by the outer membrane permeability barrier that limits access to their target sites [74,76].

Although we observed antagonism between these agents and vancomycin, both drugs have been reported to synergize with other antibiotic classes in a context-dependent manner. For example, fluoxetine enhances the activity of erythromycin and gentamicin against *Pseudomonas aeruginosa*, *Escherichia coli* and *S. aureus* [77] and has also been shown to potentiate meropenem, fosfomycin and polymyxin B against a range of Gram-negative pathogens [78]. This synergy has been attributed to inhibition of efflux, as fluoxetine exposure is associated with increased intracellular accumulation of ethidium bromide [79,80]. Similarly, amlodipine has been shown to synergize with oxacillin against *S. aureus*, although this effect appears to be strain-dependent [81]. Nevertheless, the selection of fluoxetine-resistant mutants was associated with cross-resistance to multiple antibiotics, highlighting the potential for long-term exposure to these agents to undermine antibiotic efficacy [79]. However, the accumulation of efflux-associated mutations is unlikely to confer resistance to a non-efflux substrate such as vancomycin. Therefore, the accelerated emergence of vancomycin resistance observed during co-selection with fluoxetine or amlodipine is likely independent of efflux-mediated mechanisms.

Removal of fluoxetine or amlodipine co-selection led to an almost immediate twofold reduction in vancomycin MIC. This is concerning, as the transient nature of this phenotype suggests that standard antibiotic susceptibility testing may fail to detect elevated vancomycin MICs in patients taking these medications. True vancomycin non-susceptibility is rare, with global prevalence estimates of 2.4% for vancomycin-resistant *S. aureus* and 4.3% for vancomycin-intermediate *S. aureus* [82]; by contrast, vancomycin treatment failure is relatively common, occurring in up to 50% of cases [83]. Host-associated stressors that activate the VraSR and GraSR pathways have been shown to confer transient daptomycin and vancomycin tolerance [31], supporting the concept that environmental or physiological signals can temporarily modulate antibiotic susceptibility. It is, therefore, plausible that prescription medications such as fluoxetine or amlodipine may similarly contribute to vancomycin treatment failure, although the magnitude of this effect remains unknown. Simvastatin, fluoxetine and amlodipine are among the most commonly prescribed medications in the UK, with millions of people receiving these drugs annually [35,84, 85]. Given this widespread use, the potential for these medications to influence antimicrobial susceptibility is almost certainly underappreciated.

While we did not detect activation of cell wall or membrane stress responses by simvastatin, previous studies have shown that it induces expression of *vraX*, other cell wall stress-associated genes and *mvaA* of the mevalonate pathway [53]. These discrepancies may reflect strain-specific regulation of cell wall stress response pathways [53]. We were able to further verify the effect of simvastatin on disrupting cell wall integrity through a screen of the NTML, which yielded a number of hits responsible for cell wall synthesis or turnover. Notably, loss of *sgtB* and *murE* was associated with simvastatin resistance, consistent with previous reports showing their induction upon simvastatin exposure [53]. We also observed a highly antagonistic interaction between simvastatin and oxacillin, which was able to confer transient BORSA in the MSSA strain Newman. This conflicts with previous reports of simvastatin *β*-lactam synergy [68]. Interestingly, however, a large-scale screen for antibiotic-non-antibiotic drug interactions found that atorvastatin also antagonizes *β*-lactams against *S. aureus* [86]. Similarly, the antibiotic fosmidomycin, which acts independently of the mevalonate pathway but also depletes FPP levels, protects against lysis by the cell-wall antibiotic fosfomycin [87]. Alternatively, simvastatin could be promoting biofilm production to enable growth in the presence of oxacillin. Collectively, these findings indicate that statin–*β*-lactam interactions are highly context-dependent, likely influenced by strain background, pathway activity and the *β*-lactam partner.

Polypharmacy (the concurrent use of multiple drugs) has been described as a silent pandemic, prompting initiatives focused on de-prescribing and reducing unnecessary medication burden to improve patient outcomes [88]. Our findings suggest an additional, underappreciated benefit: targeted de-prescribing may help preserve antibiotic efficacy in already vulnerable populations, strengthening the rationale for accelerating these efforts. While such initiatives are clearly important, further research is needed to fully elucidate the impact of polypharmacy on AMR and to inform optimal strategies for medication optimization.

## Supplementary material

10.1099/mic.0.001733 Supplementary Material 1.

10.1099/mic.0.001733 Supplementary Material 2.

## Abbreviations

BORSA: borderline oxacillin-resistant *Staphylococcus aureus*
FICI: fractional inhibitory concentration index
FPP: farnesyl pyrophosphate
MHB: Mueller–Hinton broth
MRSA: methicillin-resistant *Staphylococcus aureus*
MSSA: methicillin-susceptible *Staphylococcus aureus*
NTML: Nebraska Transposon Mutant Library
