# Supplementary material for: Commonly prescribed medicines antagonise anti-MRSA antibiotics and select for resistance
Source: Microbiology (Reading). 2026 Jul 2;172(7):001733. doi: 10.1099/mic.0.001733 (PMC13327645; doi:10.1099/mic.0.001733)
Supplement: Supplementary Material 1. [file mic-172-01733-s001.pdf]

Supplementary Data file

Supplementary Table. 1. Commonly used medications in the UK. Medications are categorised by class, indication, and number of annual UK prescriptions. Prescription data was obtained from the NHS Healthcare and & Prescribing Data, available at prescribemap.com.

Medication	Class	Indication	Number of prescriptions UK (in millions)
Simvastatin	Statin	Hypercholesterolemia; prevention of cardiovascular disease	11.7
Fluoxetine	Selective serotonin reuptake inhibitor	Major depressive disorder, anxiety disorder	7.5
Amlodipine	Calcium channel blocker	Hypertension and angina	39.8
Levothyroxine	Thyroid hormone replacement	Hypothyroidism	34.5
Furosemide	Loop diaretic	Oedema and hypertension	11.2
Prednisolone	Corticosteroid	Inflammatory and autoimmune conditions	6.4
Metformin	Biguanide	Type 2 diabetes mellitus	26.6
Omeperazole	Proton pump inhibitor	Gastroesophageal reflux disease, peptic ulcers	35.8

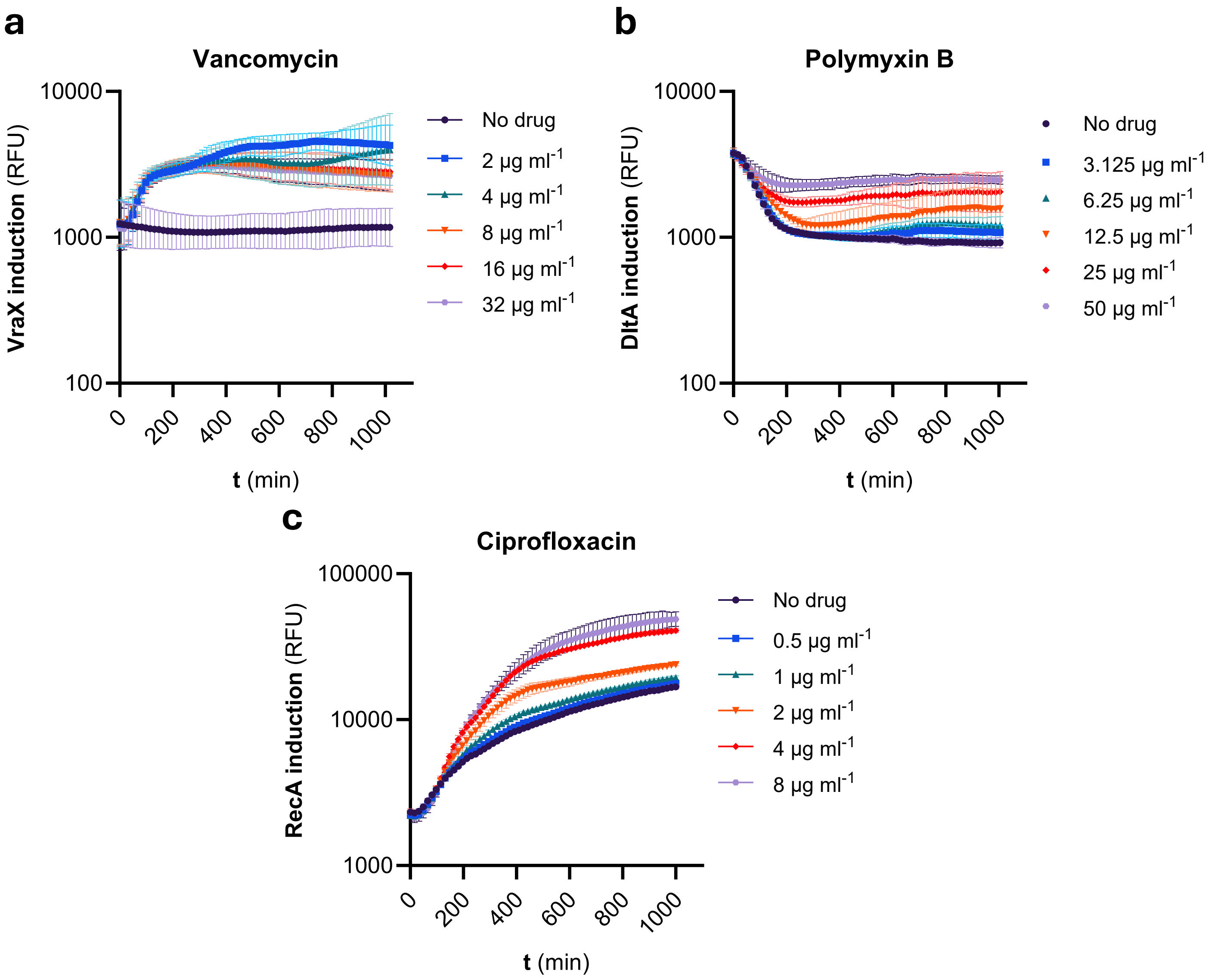

Supplementary Figure 1. GFP-fusion plasmids are induced by appropriate antibiotic controls. (a) Induction assay of p*vraX*-GFP in the presence of a 1:2 dilution series of vancomycin as determined by GFP accumulation over time. **(b)** Induction assay of p*dltA*-GFP in the presence of a 1:2 dilution series of polymyxin B as determined by GFP accumulation over time. **(c)** Induction assay of p*recA*-GFP in the presence of a 1:2 dilution series of ciprofloxacin as determined by GFP accumulation over time. All experiments were replicated in n=3 independent assays. Error bars show the standard deviation of the mean**.**

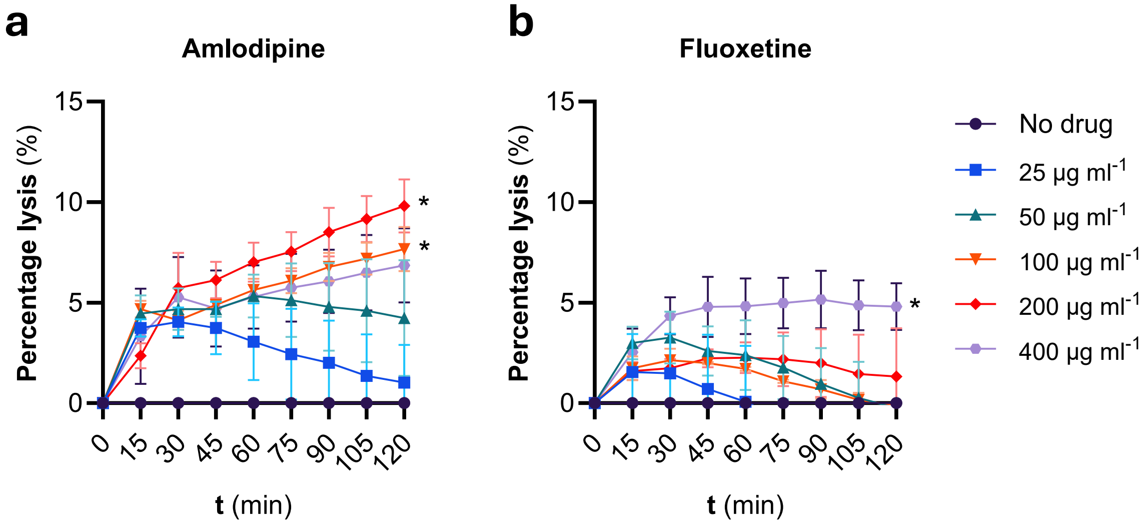

Supplementary Figure 2. Amlodipine and fluoxetine induce bacterial lysis. (a) Percentage lysis of *S. aureus* JE2 over time, exposed to a 1:2 dilution series of amlodipine. **(b)** Percentage lysis of *S. aureus* JE2 over time, exposed to a 1:2 dilution series of fluoxetine. All experiments were replicated in n=3 independent assays. Error bars show the standard deviation of the mean**.** Significance differences were determined between the no drug condition and the 400 µg/ml and 200 µg/ml treated conditions by two-way repeated measures ANOVA with post hoc Dunnett's test to correct for multiple comparisons. *$P < 0.05$.

Supplementary Table. 2. Antimicrobial activity of amlodipine and fluoxetine with and without supplementation with phosphatidylglycerol (PG). Strains are categorised by gene name (where available), indication, and simvastatin MIC.

Medication	MIC (µg/ml)	
	Without PG	With PG
Amlodipine	100	400
Fluoxetine	50	200

Supplementary Table. 3. NTML strains associated with reduced susceptibility to simvastatin.

Strains are categorised by gene name (where available), indication, and simvastatin MIC.

Strain reference	Gene name	Function	MIC (µg/mL)
JE2	WT		25
NE37	*icaA*	Biofilm	50
NE234	*cap5I*	Capsule	50
NE75	*cap1B*	Capsule	100
NE1495	*murA*	Cell wall	50
NE1693	*yycH*	Cell wall	50
NE267	*sgtA*	Cell wall	100
NE596	*sgtB*	Cell wall	200
NE217	*pknB*	Cell wall	200
NE1369	*lytH*	Cell wall	200
NE1713	*alr*	Cell wall	100
NE1099	*lyrA*	Cell wall	50
NE945	*brnQ*	Central metabolism	50
NE955	*narI*	Central metabolism	50
NE1717	*aroC*	Central metabolism	50
NE476	*fba*	Central metabolism	50
NE198	*ald*	Central metabolism	50
NE232		Central metabolism	50
NE5		Central metabolism	50
NE6		Central metabolism	50
NE233	*glpD*	Central metabolism	50
NE1896	*ipdA*	Central metabolism	100
NE16	*moaD*	Central metabolism	200
NE477	*deoD*	DNA replication/ repair/ nucleotide metabolism	50
NE1390		DNA replication/ repair/ nucleotide metabolism	50
NE243	*polA*	DNA replication/ repair/ nucleotide metabolism	50
NE246		DNA replication/ repair/ nucleotide metabolism	50
NE242	*dprA*	DNA replication/ repair/ nucleotide metabolism	50
NE277	*tdk*	DNA replication/ repair/ nucleotide metabolism	100
NE1541		DNA replication/ repair/ nucleotide metabolism	100
NE279		DNA replication/ repair/ nucleotide metabolism	100
NE1744		Hypothetical	50
NE18		Hypothetical	50
NE1402		Hypothetical	50
NE721		Hypothetical	50
NE1520		Hypothetical	50
NE1372		Hypothetical	50
NE257		Hypothetical	50
NE230		Hypothetical	50
NE4		Hypothetical	50
NE38		Hypothetical	50
NE40		Hypothetical	50
NE1809		Hypothetical	100

Strain reference	Gene name	Function	MIC (µg/mL)
JE2	WT		25
NE1861		Hypothetical	50
NE1892		Hypothetical	50
NE1831		Hypothetical	50
NE268		Hypothetical	100
NE53		Hypothetical	50
NE1800		Hypothetical	100
NE1585		Hypothetical	100
NE265		Hypothetical	100
NE1795		Hypothetical	100
NE1703		Hypothetical	100
NE50		Hypothetical	50
NE1734		Hypothetical	200
NE1909		Hypothetical	200
NE208		Hypothetical	200
NE86		Hypothetical	100
NE87		Hypothetical	100
NE258	*cls*	Membrane	100
NE209		Phage	50
NE244		Phage	50
NE699	*clpC*	Protein synthesis	50
NE289		Protein synthesis	50
NE195	*pepF*	Protein synthesis	50
NE2		Protein synthesis	50
NE896	*spxH*	Protein synthesis	50
NE1752	*rbfA*	Protein synthesis	50
NE1662	*miaB*	Protein synthesis	200
NE1269	*ohr*	Redox	50
NE1345	*menD*	Redox	50
NE1669	*nreC*	Redox	50
NE1596	*bshB2*	Redox	50
NE281		Transcription regulator	100
NE197		Transport	50
NE269		Transport	100
NE13		Transport	200
NE1890		Virulence	100

a

Fluoxetine with Vancomycin

	0	0.03125	0.0625	0.125	0.25	0.5	1	2
100	0.0516	0.0501	0.0631	0.0509	0.0503	0.0509	0.0506	0.0511
50	0.0577	0.0597	0.1733	0.1846	0.0651	0.051	0.0503	0.0512
25	0.2724	0.2156	0.165	0.2317	0.1923	0.1823	0.0498	0.051
12.5	0.352	0.3257	0.2991	0.365	0.2845	0.2652	0.0519	0.0515
6.25	0.3989	0.3496	0.3058	0.3873	0.3012	0.3131	0.1128	0.0514
3.125	0.3883	0.3498	0.3307	0.3087	0.3684	0.2795	0.1987	0.0516
1.5625	0.3824	0.3337	0.3184	0.339	0.3469	0.3101	0.0518	0.0509
0	0.3667	0.3702	0.3529	0.3704	0.36	0.3425	0.0508	0.0509

b

Simvastatin with Vancomycin

	0	0.03125	0.0625	0.125	0.25	0.5	1	2
100	0.1184	0.1348	0.1388	0.1412	0.1562	0.1371	0.1535	0.146
50	0.068	0.0724	0.0728	0.0753	0.0758	0.0734	0.0767	0.0754
25	0.1268	0.1157	0.0982	0.19	0.1334	0.081	0.0507	0.0513
12.5	0.3743	0.2832	0.2445	0.2398	0.2658	0.2418	0.118	0.0505
6.25	0.3882	0.3157	0.297	0.2742	0.2455	0.2542	0.0509	0.0504
3.125	0.3973	0.3134	0.2777	0.2769	0.268	0.2569	0.0506	0.0512
1.5625	0.3939	0.3145	0.2879	0.2895	0.2839	0.2925	0.0518	0.0503
0	0.3711	0.339	0.344	0.3391	0.345	0.3545	0.0507	0.0507

c

Amlodipine with Vancomycin

	0	0.03125	0.0625	0.125	0.25	0.5	1	2
100	0.0633	0.0632	0.1004	0.0584	0.0516	0.0502	0.05	0.0507
50	0.3039	0.305	0.3307	0.2964	0.2951	0.0534	0.052	0.0509
25	0.3473	0.3497	0.3158	0.3239	0.304	0.2369	0.0503	0.0511
12.5	0.3729	0.3417	0.3104	0.3043	0.3035	0.295	0.05	0.0507
6.25	0.3625	0.3445	0.301	0.2764	0.2673	0.2867	0.0503	0.0493
3.125	0.3651	0.3363	0.3276	0.289	0.2761	0.2881	0.0616	0.0517
1.5625	0.3831	0.3587	0.3125	0.2971	0.3156	0.3248	0.1083	0.0529
0	0.3449	0.3536	0.3421	0.3682	0.3541	0.3458	0.0505	0.0509

d

Fluoxetine with Daptomycin

	0	0.03125	0.0625	0.125	0.25	0.5	1	2
100	0.0556	0.0529	0.0536	0.0537	0.0527	0.0527	0.0537	0.0544
50	0.1601	0.1267	0.1166	0.1191	0.1223	0.1215	0.1218	0.0544
25	0.4185	0.3686	0.3384	0.3046	0.349	0.3191	0.3205	0.0548
12.5	0.5281	0.4279	0.3982	0.4107	0.4346	0.4096	0.3808	0.0541
6.25	0.4932	0.4847	0.4773	0.4268	0.4718	0.4409	0.394	0.0554
3.125	0.5041	0.4626	0.4588	0.4785	0.4877	0.4655	0.3656	0.0572
1.5625	0.5673	0.455	0.4178	0.4125	0.4621	0.417	0.3846	0.0563
0	0.519	0.4787	0.4824	0.4659	0.4887	0.4355	0.3093	0.0561

e

Simvastatin with Daptomycin

	0	0.03125	0.0625	0.125	0.25	0.5	1	2
100	0.1279	0.169	0.1703	0.1603	0.1831	0.1758	0.1648	0.1537
50	0.0622	0.0658	0.0609	0.0662	0.0647	0.0635	0.0658	0.0652
25	0.3489	0.297	0.2597	0.256	0.2195	0.2103	0.0555	0.0543
12.5	0.4743	0.415	0.3359	0.3632	0.368	0.3537	0.2696	0.0545
6.25	0.4715	0.3902	0.3086	0.3339	0.3418	0.3272	0.2324	0.0546
3.125	0.4675	0.3859	0.3616	0.3623	0.3425	0.377	0.3363	0.0563
1.5625	0.4727	0.4188	0.3975	0.4055	0.4068	0.3917	0.2059	0.0556
0	0.449	0.4572	0.4658	0.4457	0.4597	0.3965	0.2103	0.0562

f

Amlodipine with Daptomycin

	0	0.03125	0.0625	0.125	0.25	0.5	1	2
100	0.0744	0.0574	0.065	0.0591	0.0667	0.0587	0.0594	0.0558
50	0.4147	0.4444	0.4321	0.3802	0.4293	0.4468	0.3384	0.0542
25	0.4093	0.4204	0.4295	0.4381	0.4539	0.3625	0.0936	0.0545
12.5	0.4653	0.4232	0.4482	0.4297	0.4118	0.4016	0.1944	0.0534
6.25	0.4469	0.4567	0.419	0.3898	0.3495	0.3482	0.1115	0.0541
3.125	0.5109	0.4784	0.447	0.4779	0.4156	0.4154	0.1236	0.0559
1.5625	0.463	0.5043	0.4846	0.5464	0.5329	0.4938	0.059	0.0554
0	0.4224	0.3764	0.3512	0.3604	0.4362	0.3312	0.0583	0.0574

Supplementary Figure 3. Fluoxetine, amlodipine and simvastatin antagonise anti-MRSA antibiotics. (a, b, c) Checkerboard broth microdilution assay showing the antagonistic interaction between vancomycin and **a)** fluoxetine, **b)** simvastatin, and **c)** amlodipine against *S. aureus* JE2. **(d, e, f)** Checkerboard broth microdilution assay between daptomycin and **a)** fluoxetine, **b)** simvastatin, and **c)** amlodipine against *S. aureus* JE2. Green cells indicate growth, and white cells indicate no growth.
